# Supplementary material for: Association between oral microbiome and seven types of cancers in East Asian population: a two-sample Mendelian randomization analysis
Source: Front Mol Biosci. 2023 Nov 21;10:1327893. doi: 10.3389/fmolb.2023.1327893 (PMC10702768; doi:10.3389/fmolb.2023.1327893)
Supplement: Supplementary file 1 [file DataSheet1.ZIP › Supplementary Materials/MR plots of saliva/saliva═╝/ovarian cancer/pheno.1269_to_ovarian cancer_forest.pdf]

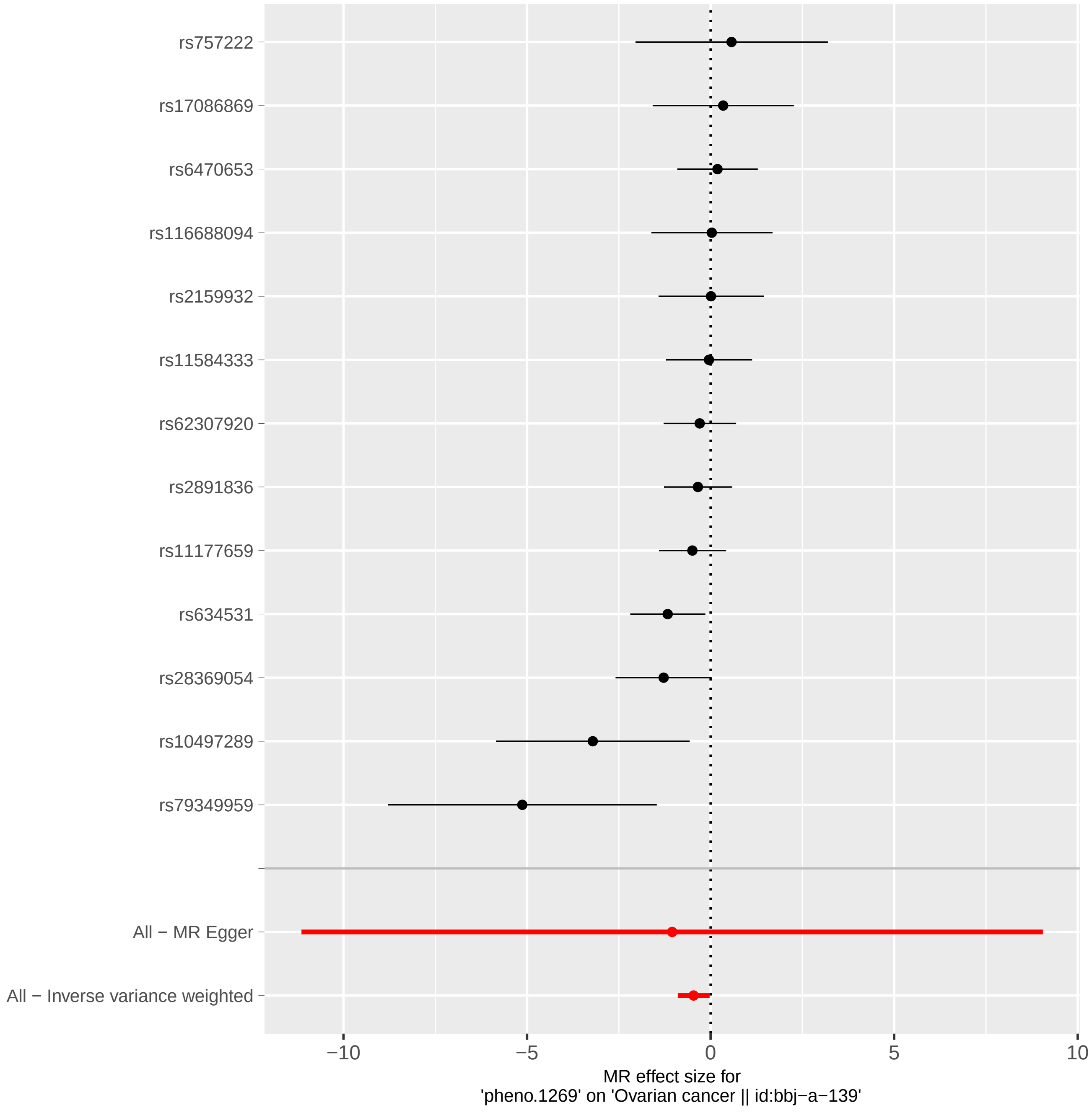

rs757222
rs17086869
rs6470653
rs116688094
rs2159932
rs11584333
rs62307920
rs2891836
rs11177659
rs634531
rs28369054
rs10497289
rs79349959
All − MR Egger
All − Inverse variance weighted
−10
−5
0
5
10
MR effect size for
'pheno.1269' on 'Ovarian cancer || id:bbj−a−139'
